# Limited Accuracy of Colour *Doppler* Ultrasound Dynamic Tissue Perfusion Measurement in Diabetic Adults

**DOI:** 10.1371/journal.pone.0168905

**Published:** 2016-12-29

**Authors:** Felix Stoperka, Claudia Karger, Joachim Beige

**Affiliations:** 1 Dept. Nephrology and Kuratorium for Dialysis and Transplantation (KfH) Renal Unit Hospital St. Georg, Leipzig, Germany; 2 Martin-Luther-University Halle/Wittenberg, Halle, Germany; Shenzhen institutes of advanced technology, CHINA

## Abstract

Dynamic tissue perfusion measurement (DTPM) is a pre-described and available method in pediatric ultrasound to quantify tissue perfusion in renal *Doppler* ultrasound by particular video analysis software. This study evaluates DTPM during single and between repeated visits after 6 months, calibrates repeated DTPM within different region of interest (ROI) and compares DTPM with kidney function markers in adult patients with early diabetic nephropathy (n = 17). During repeated measurements, no association of readings at the same patients in the same (n = 3 readings) as well as repeated visit (n = 2 visits) could be retrieved. No association between DTPM, MDRD-GFR, albuminuria, age and duration of diabetes was observed. These negative results are presumably related to inconsistency of DTPM due to non-fixed ROI position as could be shown in calibrating series. Further development of the method should be performed to enable reproducible DTPM readings in adults.

## Background

Assessment of renal function and early diagnosis of chronic kidney disease (CKD) requires sensitive and robust tools to characterize excretory kidney function, albuminuria, and structural organ changes. Assessment of perfusion by *Doppler* ultrasonography (US) is used to describe changes of renal vasculature and succeeding disease course. Because only limited information concerning single vessels can be drawn from *Doppler* measurements alone, some alternative methods to characterize parenchyma perfusion can be used. Beside contrast enhanced sonography [1], dynamic tissue perfusion measurement (DTPM) by a colour-pixel quantifying picture analysis (*Pixelflux*^®^, Germany) was introduced and has demonstrated the potential to describe perfusion in renal cortex in children kidneys, children transplants and other organs. These authors described correlations between perfusion quantitative measures and renal transplant function. However, these measurements were done in yearly subsequent investigations of paediatric renal graft recipients without standardized data concerning repeatability during the same visit [2, 3]. Within the respective paper, a huge DTPM difference was displayed between the overall cohort during year 1 and 5 (0.4cm/sec) and a subset of patients with grafts surviving longer than 9 years. Further issues included DTPM differences between normal and inflammatory hyperperfused bowel segments in Crohn disease and ulcerative colitits, in lymphadenitis of infectious mononucleosis and Hashimoto thyreoiditis [4–6]. The inaugurating group was also able to correlate kidney perfusion measurements to MAG3 scintigraphy [5] and renal function represented by serum creatinine levels. The promising and broadly published *Pixelflux*^®^ method was primarily developed in the environment of paediatric or veterinary medicine [7–10] and there is no information available about the stability of measurements in long-term investigational series of adult patients. *Lubas* et al. reported cross-sectional DTPM data in 17 consecutive adult patients with CKD. They described a significant cross-sectional association between distal perfusion index (DPI) and GFR_CKD-epi_ and age, but not between proximal perfusion index (PPI) and anthropometrical and kidney function measures [11]. *Rosenbaum* et al. showed that this method could be used to evaluate organ perfusion but in their study no association between perfusion indices and pathological characteristics of renal cell carcinoma was found [12].

Diabetic nephropathy (DN) during early disease stages (CKD 1) is theoretically characterized by enhanced renal perfusion in frame of hyperfiltration [13]. It is not known whether this phenomenon can be measured by DTPM or any parenchyma perfusion methods. We therefore aimed to standardize DTPM in the setting of early DN in order to develop a tool for further monitoring of vasculature—structural changes during that disease. As a first step, repeated DTPM during a single visit and between repeated visits should be compared with each other. Markers of kidney function (glomerular filtration rate, GFR) by the Modification of Diet in Renal Disease study (MDRD) formula) [14] were obtained to perform association studies between DTPM and renal function.

## Materials and Methods

### Patient population, data acquisition and handling, patient consent

We included patients of our out-patient diabetes center during routine appointments. Inclusion criteria were age 18–80 years and GFR_MDRD_ > 60mL/min. Exclusion criterion was non-ability to understand or non-agreement with written informed consent. The study was approved by the Ethical Review Board (ERB) of the Saxonian Association of Physicians, Dresden, Germany (# EK-BR 88/13-1). Participant′s written informed consent was filed in study records.

We performed 2 consecutive US visits during a period of 6 months as described below.

DTPM and anthropometrical data, patient history, and laboratory data were exported to a pseudo-anonymous Microsoft Excel^®^ file and merged to a SPSS^®^ vs 13 dataset. This final dataset comprised 17 patients and was subjected to further analysis. A preliminary raw data set necessary to replicate core results was uploaded to a public repository (https://figshare.com/articles/Excel_Dataset/4232933).

### Statistical analysis

Data are presented as means ± standard deviation or proportions. Continuous variables were compared by two-sided Student’s t test. Categorical data were assessed by χ^2^ statistics. Association studies were performed by ANOVA. Same variables at repeating time-points were compared by paired t–tests.

### Ultrasound technique

To reach a maximum of reproducibility, as much as possible preset US conditions were fixed. All colour *Doppler* examinations were performed with a 5 to 2 MHz curved array probe and Philips ultrasound equipment (HD15, Philips, Hamburg, Germany 2009). Colour gain was set between 51 and 69 (median 63) but could not be fixed at one single value because of the orthotopic kidney position, obesity and different perfusion status. Pulse repetition frequence (PRF) was allowed to be changed to avoid aliasing and was considered by the analysis program. All patients were examined in a ventral position [15].

2 or 3 video sequences per patient were recorded for 3 seconds using longitudinal sections of the right kidneys and used for perfusion calculation. Between the recordings the transducer was moved and after a few seconds the next sequence with a reproduced transducer position was recorded. Because Pixelflux^®^ implicates no adaptation to heart cycles we decided to fix the time of all video sequences to 3 seconds. Such standardization enables highest intra-individual stability while jeopardizing the comparability between patients. *Scholbach* et al. used the same duration and method [16].

Sequences were uploaded to DTPM analysis software which calculates the number of red and blue pixels within a video sequence to estimate perfusion (*Pixelflux*^®^ *Scientific*, version 11_08_27, Chameleon Software GmbH, Münster, Germany).

The formula used by Pixelflux^®^ to calculate the perfusion intensity is [16]:

$$I\;\left[cm/s\right]=\frac{\text{A }\left[cm^{2}\right]\;\times \;\left|v\right|\left[cm/s\right]\;}{{\text{A}}_{\text{RoI }}\left[cm^{2}\right]}$$

**Fig 1:**
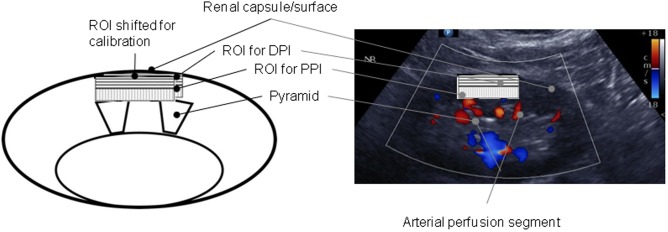
Schematic (left) and in vivo (right) positioning of the ROI in longitudinal US sections of right kidneys. The ROI parallelogram was situated above medullary pyramids and under the capsule but between the middle of adjacent pyramids corresponding to a typical arterial perfusion segment. The proximal ROI area was considered for PPI and the distal area for DPI.

The mean velocity (v) of all pixels and the area (A) are multiplied and divided by the area of the whole ROI. The calculation of PPI and DPI uses the same formula but different regions of interest (ROI). To position the ROI to be analyzed, we used a method described by *Scholbach* et al. (Fig 1) [3] which is incorporated in *Pixelflux*^®^ analysis software. ROI included the area between the middle of medullary pyramids (usually corresponding to the side limits of one renal artery segment), the top of pyramids and the surface (capsule) of the kidney. Of note, to identify this area, video sequences of the pulsating vessels were regarded. Fig 1 represents a typical ROI position in still picture (further still picture example can be seen on https://figshare.com/articles/Example_ROI_still_picture/4232900 and a video sequence on https://figshare.com/articles/Video_sequence_to_place_ROI/4232921.

The area displayed by vertical lines in the proximal 50% of ROI was considered to correspond to the proximal perfusion index (PPI) and the horizontal lines to the distal perfusion index (DPI). To calibrate the variability of total perfusion depending on the positioning of ROI we examined three healthy subjects (age 23, 25 and 29) with different approaches of positioning. First, the standard position following *Scholbach* was used and second, the ROI was displaced by 10% of the ROI′s height towards the medulla to read more intensive perfusion.

## Results

The mean results of proximal and distal DTPM coefficients are given in Table 1.

**Table 1:** Mean Results of DTPM measures in subsequent US visits.

	visit 1 (n = 17)	visit 2 (n = 16)	P
	Mean ± SD		
PPI (cm/sec)	0.56 ± 0.38	0.16 ± 0.14	0.024
DPI (cm/sec)	0.12 ± 0.17	0.02 ± 0.05	0.001

In the majority of US investigations, DPI could not be retrieved properly because of reduced peripheral perfusion which was not detectable by the ultrasound machine. Therefore, further DTPM analyses have been performed solely by PPI.

**Fig 2:**
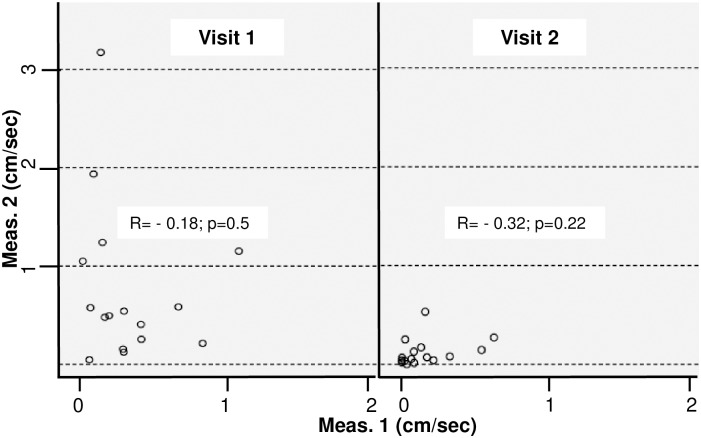
Correlation of 2 subsequent PPI measurements during visit 1 (a, left panel) and visit 2 (b, right panel).

The distribution of 2 proximal DTPM (PPI) coefficients in each of 2 single visits is given in Fig 2a) and 2b). There was no significant association of repeated measures.

Descriptive population analysis after dichotomizing for subgroups with mean high or low PPI is given in Table 2.

**Table 2:** Population characteristics by equal-size groups of high or low PPI at visit 1.

Visit 1	PPI < 0.42	PPI > 0.42	Total	p
	n = 9	n = 8	n = 17	
	Mean ± SD			
PPI visit 2 (cm/sec)	0.144 ± 0.117	0.183 ± 0.175	0.164 ± 0.143	0.59
Age (years)	69.3 ± 5.23	67.3 ± 6.77	68.5 ± 5.36	0.89
BMI (kg/m^2^)	31.5 ± 5.45	30.4 ± 4.20	30.8 ± 3.9.7	0.53
BP sys (mm/Hg)	145 ± 21	143 ± 20	144 ± 19	0.57
Male gender (n, %)	4, 57	6, 60	10, 59	0.65
Diabetes duration	13.2 ± 7.14	14.7 ± 9.47	14.1 ± 8.55	0.43
GFR_MDRD_ baseline (ml/min/1.73m^2^)	79.5 ± 16.4	87.7 ± 21.3	83.2 ± 18.5	0.97
GFR_MDRD_ visit2 (ml/min/1.73m^2^)	83.2 ± 19.4	87.4 ± 19	85.3 ± 17.8	0.63
HbA1c baseline (%)	6.43 ± 0.87	7.06 ± 1.16	6.73 ± 1.03	0.22
HbA1c visit 2 (%)	6.82 ± 0.66	6.90 ± 1.23	6.86 ± 0.94	0.81
MiAu baseline (mg/l)	65.5 ± 115.2	16.5 ± 24.6	36.7 ± 74.7	0.21
MiAu visit 2 (mg/l)	132.6 ± 229.5	18.2 ± 22.4	65.3 ± 148.4	0.13
Insulin dependent (n, %)	3, 43	8, 80	64,7	0.15

There was no association of variables with PPI at visit 1 (Table 3) or visit 2 (data not shown).

**Table 3:** Linear regression analysis between variables with PPI at visit 1.

Variable	Regress. coeff. B	P	95% CI	
Age	0.132	0.111	-0.048…	0.311
BMI	-0.100	0.111	-0.235…	0.036
BP sys	-0.016	0.312	-0.055…	0.023
BP dia	0.054	0.120	-0.022…	0.130
Gender	-0.302	0.340	-1.076…	0.472
Diabetes duration	0.034	0.238	-0.034…	0.103
GFR_MDRD_ baseline	0.026	0.216	-0.024…	0.076
GFR_MDRD_ diff.	0.033	0.148	-0.018…	0.084
HbA1c baseline	-0.066	0.611	-0.402…	0.269
MiAu baseline	-0.002	0.274	-0.007…	0.003

When total DTPM without regard to proximal or distal perfusion was analyzed, no correlation between DTPM and ROI position was found (Table 4).

**Table 4:** Results of serial calibration measurements. Association between total DTPM and ROI position in 3 individuals.

		Total DTPM (cm/sec)			*Mean*
		Invest. #1	Invest. #2	Invest. #3	
**Subj. A**	Standard ROI pos.	0.256	0.711	0.334	*0*.*434*
	Displaced ROI pos.	0.776	1.212	0.876	*0*.*955*
	*Rel*. *difference (%)*	*203*	*70*	*162*	*145*
**Subj. B**	Standard ROI pos.	0.113	0.144	0.070	*0*.*109*
	Displaced ROI pos.	0.271	0.437	0.336	*0*.*348*
	*Rel*. *difference (%)*	*140*	*203*	*380*	*241*
**Subj. C**	Standard ROI pos.	0.123	0.159	0.197	*0*.*160*
	Displaced ROI pos.	0.275	0.247	0.771	*0*.*431*
	*Rel*. *difference (%)*	*124*	*55*	*291*	*157*

## Discussion

With the present investigation, we aimed to standardize and evaluate ultrasound DTPM measurement in the setting of adult patients to characterize developing diabetic nephropathy. However, although we used highly standardized examination conditions (same investigator, same US preset, same patient position, same ROI position) and contemporary US machinery we were not able to reproduce our measurements either during subsequent readings within one visit as well as between subsequent visits after 6 months. In a calibrating attempt with 6 measurements at 2 different ROI positions within the perfusion video loops we could show that one of the crucial points of the methodology is ROI positioning yielding different results after serial DTPM readings (Table 4). Although all proximal shifted ROI′s (i.e. directed to a region with higher perfusion) showed higher perfusion indices, the increase between immediately following readings showed random differences including more than 200% deviation when different individuals were compared. Therefore, following these difficulties we think that the method has to be developed and enhanced concerning this particular point of ROI positioning. Maybe an automated (picture-based) positioning is the better solution compared to the present investigator-driven approach.

This is the first study using such a serial and calibrating evaluation design. In the pioneering studies from *Scholbach* et al., no serial comparisons of DTPM measures were shown, while the standard deviations of same kidneys (paediatric transplants) seemed to be substantial lower compared to our adult orthotopic results. However, even in their data, about 200% perfusion difference between year one and two after transplant was detected and not linear correlated with renal function. Children transplant kidneys normally do show a much more intensive perfusion pattern based on not only large, but predominantly small vessels. In opposite, older kidneys are subjected to large changes in perfusion measurements following small changes in position of large vessels. The vessel—transducer—angle, which impacts on perfusion quantification, is different compared to orthotopic kidneys, although we studied patients in a ventral position, what is considered optimal for renal ultrasound and tried to employ a standard 90 degree skin—probe angle [5]. Angle differences may impact on the DTPM repeatability because they influence velocity measurement. Another unsolved issue belongs to the number of included heart cycles in perfusion calculation. Because the number of counted pixels is highest during the systolic phases, the number of systoles needs to be standardized. Pixelflux in it′s current stage does not provide heart-cycle normalization and is usually based on a 3-second reading duration. This duration provides intra-individual but not inter-individual and visit-to-visit stability of included heart cycles, because they may change over time [2–4]. A lower signal-noise ratio in adults compared to children is based on a less dense vascular bed in individuals of advanced age. Analysing the signal-noise ratio and adjusting for differences between children and adults would require to quantify the artefact-threshold of *Doppler* signals, currently not provided in Pixelflux^®^ analysis.

Within the only available adult study, serial readings were not performed as well. That group around *Lubas* notified the same phenomenon as our group, in particular difficulties to obtain DPI values. Therefore, in our and their study, only PPI were considered for further investigation.

Of course, our methodology may inherit weaknesses or incorrectness in addition to the question of positioning. Colour gain adjustment must have been adapted for better perfusion image, but in an acceptable narrow range. PRF adjustment was done but implemented into the software algorithm. Looking at the primary study hypothesis—association between disease stage surrogates as albuminuria, age, duration of diabetes and renal function—no association with either DTPM index was found. DTPM results after 6 months (visit 2) showed substantial lower values beside random change, which could not be associated with decreased renal function. This negative result in terms of function association and repeatability, however, has to be interpreted with reluctance because of the failed evaluation series. A second issue belongs to the different methods of renal function assessment in children by a clearance formula [17] vs. adults where MDRD formula [14] applies estimation by age and gender.

In summary we evaluated Pixelflux^®^ DTPM method in the setting of adult kidney ultrasound the first time by serial and multiple measurements. We were not able to reproduce the results taken in children and transplant kidneys presumably due to pictorial uncertainty of ROI position and presumably missing standardization of vessel-transducer angle, heart cycle reading and signal-noise normalization. For routine investigations in adults, Pixelflux^®^ DTPM can not be used in it′s present form. This valuable method should be further developed in terms of the discussed shortcomings to make use of the meaningful perfusion intensity calculation on the basis of better standardized perfusion reading.
